# Associations between sleep difficulties and health outcomes in treatment-seeking veterans

**DOI:** 10.1093/occmed/kqad094

**Published:** 2023-10-20

**Authors:** N Molloy, D Murphy

**Affiliations:** King’s Centre for Military Health Research (KCMHR), King’s College London, London SE5 9RJ, UK; Combat Stress, Tyrwhitt House , Leatherhead KT22 0BX, UK

## Abstract

**Background:**

Sleep disturbance in UK Armed Forces personnel appears to be frequent due to factors such as hostile sleeping environments and can persist even once they have transitioned into civilian life. Despite this, there is currently very limited literature surrounding the prevalence and associated factors of insomnia disorder among UK veterans.

**Aims:**

This study aimed to expand knowledge of the prevalence and associated demographic, military, health and functional outcomes with probable insomnia disorder within a clinical sample of veterans.

**Methods:**

Treatment-seeking veterans from a national UK mental health charity were invited to complete a questionnaire including socio-demographic, military, health and well-being questions.

**Results:**

Of the sample, 489 (43%) completed the questionnaire. Seventy per cent of the sample reported having probable insomnia disorder. Having probable insomnia disorder was significantly associated with being younger and having physical health problems. Moreover, unadjusted models found associations between probable insomnia disorder and common mental health difficulties, obsessive–compulsive disorder and complex post-traumatic stress disorder.

**Conclusions:**

The results suggest that many UK veterans with physical and mental health difficulties experience co-morbid insomnia disorder. Therefore, it is important that clinical services are aware of this prevalence and use targeted interventions to reduce the frequency of insomnia disorder in this population.

## Teaser

Sleep difficulties are common in Armed Forces personnel and can persist even once they leave the military and transition to civilian life. Despite this, there is little research investigating insomnia disorder in UK veterans. This study found 70% of a sample of treatment-seeking veterans reported a probable insomnia disorder. Those who were younger and had poor physical health were significantly more likely to report probable insomnia disorder.

**Key learning points**

**What is already known about this subject:**

- Armed Forces personnel often have disrupted sleep patterns while deployed due to factors like hostile sleeping environments, often persisting once transitioned into civilian life.
- Well-established links between sleep difficulties and poor physical and mental health have been explored both within the general population and Armed Forces samples.
- Despite this, little research has been conducted into insomnia disorder in Armed Forces personnel, and there is currently no prevalence data within UK veteran samples.

**What this study adds:**

- Seventy per cent of our sample of veterans reported a probable insomnia disorder, indicating it is highly prevalent within treatment-seeking veterans within the UK.
- A probable insomnia disorder was also significantly associated with having poor physical health and being younger.

**What impact this may have on practice or policy:**

- This study highlights that probable insomnia disorder is highly prevalent in treatment-seeking veterans and those who are already seeking help for physical and/or mental health difficulties may also need to be assessed for disturbed sleep to allow for sleep interventions alongside other treatment they may be receiving.
- There also may be a potential of promoting healthy sleeping patterns while serving in the Armed Forces and post-deployment for veterans to carry this on once transitioned to civilian life.

## Introduction

While serving in the Armed Forces, personnel must remain alert due to high-risk environments and the risk to themselves and their units. However, prolonged restricted sleep time has been linked to significant consequences like increased accidents, cognitive dysfunction and lapses of attention [1].

However, there is no current data on the prevalence of insomnia disorder in the UK Veteran population [2]. The main symptoms of insomnia disorder include difficulty with sleep initiation, duration, quality or consolidation despite opportunity, and circumstances for sleep which cause some form of daytime impairment [3]. The US military reported that 26% of veterans reported insomnia disorder [4]. Similarly, several UK-based studies have found around a third of UK adults have insomnia disorder symptoms [5–7].

It has been hypothesized that insomnia disorder can start while serving in the Armed Forces, and then continue into veteran life, making reintegration into civilian life more challenging [8]. Research from the USA has highlighted that there may be a stigma while serving surrounding admitting a greater need for sleep [9]. This could have serious implications as this may prevent those raising the issue until it becomes chronic [10]. It is well accepted that sleep disturbances that begin during service may persist even once transitioned into civilian life, with one study finding almost 9 in 10 service personnel and veterans reporting being classed as poor sleepers post-deployment [8, 10].

There is a well-established link between sleep disturbance and mental health difficulties, both within the general population [11] and Armed Forces personnel [12]. A large proportion of those with mental health difficulties such as depression are at a high risk of reporting co-morbid insomnia disorder; one study found 83% of depressed participants reported at least one insomnia disorder symptom compared to only 36% of non-depressed participants [13]. Investigations into serving personnel immediately and 6-month post-deployment found sleep problems were significantly associated with alcohol use disorders and common mental health difficulties (CMD) [14].

Additionally, there is increasing evidence that disturbed sleep is linked to poor physical health outcomes both within and outside of the UK Armed Forces [13, 14]. There are strong links between sleep disturbances and issues like obesity and diabetes [15]. Despite this, research in the USA found that veterans specifically may have undiagnosed or under-reported sleep conditions due to sleep disturbance being a symptom of another psychological or medical condition rather than a focus as a primary disorder [10].

Overall, research in the UK Armed Forces has tended to focus more on the prevalence of mental health difficulties, and despite sleep disturbance being a key symptom of many of these disorders, a limited amount of insomnia disorder data is available [14]. Furthermore, there is little research among the veteran population despite the co-morbidities and the long-term persisting consequences of disturbed sleep which is likely to continue after leaving the Armed Forces. Therefore, this study aimed to investigate the prevalence of probable insomnia disorder within a sample of treatment-seeking veterans and to explore the associated socio-demographic, military, health and functional outcomes associated with probable insomnia disorder to identify who may be more at risk.

## Methods

Participants were recruited from Combat Stress, a UK Veterans mental health charity. The study used a cross-sectional design.

Participants were all treatment-seeking veterans accessing services from across the UK. Participants were randomly sampled from the Combat Stress Case Management System in which they had (i) received support from Combat Stress over a 1-year period, (ii) consented to be contacted for research purposes (iii) and provided a contact e-mail address. Within this sample, treatment-seeking was defined as an individual who had attended at least one session with Combat Stress. Individuals were defined as veterans if they had completed a minimum 1-day paid employment in the UK Armed Forces. A random subsample of 20% equated to 1147 veterans. One hundred and fifty eight of these had an invalid e-mail address, leaving 989 veterans who were contacted by the research team.

Participants received a 10-page questionnaire which was split into eight sections. This included (1) About you, (2) Your Military History, (3) Questions About Your Social Network, (4), Questions About Your Gambling and Drinking Habits (5) Questions About Your Health, (6) Questions About Your Obsessions and Compulsions, (7) Questions About Symptoms Related to a Stressful Event and (8) Questions About Life Growing Up.

Data collected on socio-demographic characteristics included, sex, age, ethnicity, employment status and relationship status. Military characteristics consist of service branch, rank, reason for leaving the Armed Forces and whether they were an early service leaver.

Sleep was measured using the eight-item Sleep Condition Indicator [16], a scale that measures sleep problems against criteria for probable insomnia disorder. Eight questions were asked regarding the last month, with scores ranging on a 5-point Likert scale (0–4). An example stem question is ‘Thinking about a typical night in the last month … *how would you rate your sleep quality*’. Total scores are reported between 0 and 32 whereby a score of 16 or less indicates insomnia disorder and a higher score suggests better sleep. The Cronbach’s alpha for this study was 0.91, which is very good.

Loneliness was measured using the three-item UCLA Loneliness Scale (UCLA-3) [17], which measures three dimensions of loneliness; relational connectedness, social connectedness and self-perceived isolation. Scores from 3 to 5 are classified as ‘not lonely’ and scores of 6–9 are categorized as ‘lonely’. Perceived Social Support was measured using the Oslo Social Support Scale-3 (OSSS-3) [18]. Scores can be categorized as poor (3–8), moderate (9–11) and strong (12–14) social support. Well-being was measured using the short Warwick–Edinburgh Mental Well-being Scale whereby a score of below 19.5 indicated a case of low well-being [19].

Other health factors included physical health and CMD. Physical health was measured using the 15-item Patient Health Questionnaire (PHQ-15) [20]. Higher scores indicate more severe symptoms, 0–4 indicate minimal symptoms and scores between 15 and 30 represent high somatic symptoms severity. The 12-item General Health Questionnaire (GHQ-12) [21] was used to measure CMDs such as depression and anxiety. A score of 4 or higher indicates a possible presence of CMDs.

Questions related to Alcohol Use were measured using the Alcohol Use Disorder Identification Test [22], whereby a score of 8+ indicates hazardous alcohol use and 16+ harmful alcohol use. Obsessions and Compulsions were measured using the Yale–Brown Obsessive Compulsive Scale (Y-BOCS) [23]. Scores on the Y-BOCS can be categorized into mild (8–15), moderate (16–23), severe (24–31) and extreme (32–40). Moreover, the Dimensions of Anger Reactions-5 (DAR-5) [24] measured difficulties with anger. A score of 12 or higher indicates possible anger difficulties.

Post-traumatic stress disorder (PTSD) and complex post-traumatic stress disorder (CPTSD) were measured using the International Trauma Questionnaire (ITQ) [25, 26]. Participants had to consider their most traumatic event and how much they have been bothered by core symptoms in the last 6 months. Six items measured PTSD symptoms (re-experiencing, avoidance and sense of threat clusters) and six items measured disturbance in self-organization symptoms (functional impairment, affective dysregulation and negative self-concept clusters). A Likert scale of five points measures each item from 0 (not at all) to 4 (extremely). ITQ total scores can range from 0 to 24. Diagnostic criteria for PTSD require a score of two or more for at least two symptoms for each of the three clusters. For diagnostic criteria of CPTSD, one must satisfy the criteria for PTSD plus scoring two or more for at least one symptom for each of the three clusters.

At first, data were collected via Survey Monkey, an online survey creator and distributor between August and September 2020. Participants received five e-mail invitations over a period of 6 weeks. After this, all non-responders (*n* = 692) were sent a paper questionnaire via postal mail out in October 2020.

The first stage of the analysis was to describe the sample characteristics by calculating the prevalence rates of the socio-demographic variables and the prevalence of sleep disturbance. Following this, linear regression models were fitted to explore the associations between socio-demographic variables and sleep disturbance. These were then adjusted by adding in all the socio-demographic variables into the model. Multiple linear regression models were then fitted to explore the associations between sleep disturbance and functional impairment and health outcomes. These models were adjusted for the other functional impairment or health outcome variables, respectively. Due to previous research findings, responders in this study were significantly more likely to be older than non-responders; survey weights were used to account for this [27]. All analyses were conducted using STATA Version 13.0 [28].

Approval for the study was granted by the Combat Stress Research Committee (ref. pn2020). When providing consent, participants agreed that anonymized survey responses could be used for research.

## Results

Table 1 describes the socio-demographic and military characteristics of the sample. In total, 989 veterans were contacted, and 428 participants responded making a final response rate of 43%. Overall, 70% of the sample reported having difficulties with sleep, indicating a likely insomnia disorder whereas just under a third of the sample did not report having difficulties with sleep (30%).

**Table 1.** Sample descriptors

Variable	*n* (%)
Gender	
Male	417 (97)
Female	11 (3)
Age	
<35	46 (11)
35–44	86 (20)
45–54	146 (34)
55+	150 (35)
Ethnicity	
White	379 (95)
Ethnic minority	21 (5)
Employment status	
Working or retired	223 (56)
Not working	173 (44)
Relationship status	
In relationship	264 (66)
Not in relationship	133 (34)
Housing status	
Fixed address	361 (91)
No fixed address	36 (9)
Service branch	
Army	353 (82)
Naval Services	47 (11)
RAF	28 (7)
Last rank	
Officer	44 (11)
Other ranks	349 (89)
Early service leaver	
No	368 (96)
Yes	17 (4)
Reason for leaving military	
Voluntary	213 (55)
Non-voluntary/medical	175 (45)
Sleep disorder	
No	115 (30)
Yes	269 (70)

*Notes: n* = 428. As a result of missing data, some frequencies do not sum to 428.

Most of the samples were male (97% versus 3% female). Most (95%) had a White ethnicity, whereas 5% identified as an ethnic minority. Just over half of the sample were working or retired (56%) with 44% not working. Many were in a relationship (66%), and most were living at a fixed address (91% versus 9% not at a fixed address).

Moreover, most of the sample had served in the British Army (82%), with the Royal Naval Services making up 11% of the sample and 7% having served in the Royal Air Force. Eleven per cent had served in the rank of an Officer, whereas most served in Other Ranks (89%). Most veterans were not Early Service Leavers (96%) whereas a small proportion was Early Service Leavers (4%) and just over half were voluntary discharges (55%) and the remaining 45% were non-voluntary or medical discharges.

Next, a multiple linear regression model was run to look at associations between probable insomnia disorder, socio-demographic and military factors. Table 2 presents the outcome of this analysis in more details. There was a significant association between probable insomnia disorder and those in the age band of 35–44 years old (−2.52, 95% CI −4.09, −0.95, *P* < 0.01). No other significant associations were found between probable insomnia disorder and socio-demographic and military characteristics in the adjusted model. In the unadjusted model, there was a significant association between probable insomnia disorder and those not in a relationship (−1.43, 95% CI −2.81, −0.06, *P* < 0.05); however, this did not remain significant in the adjusted model.

**Table 2.** Associations between socio-demographic and military variables and sleep

	Mean (SE)	Unadjusted	95% Confidence Interval	Adjusted	95% Confidence Interval
Age					
<35	13.24 (1.50)	1.00		1.00	
35–44	10.77 (0.96)	−2.47 ^**^	−3.37, −1.58	−2.52 ^**^	−4.09, −0.95
45–54	12.45 (0.81)	−0.80	−2.14, 0.54	−0.96	−3.03, 1.11
55+	12.99 (0.78)	−0.25	−1.23, 0.73	−0.54	−2.33, 1.25
Relationship status					
In a relationship	12.92 (0.56)	1.00		1.00	
Not in Relationship	11.49 (0.84)	−1.43 ^*^	−2.81, −0.06	−0.80	−2.42, 0.80
Ethnicity minority					
White	12.45 (0.47)	1.00		1.00	
Ethnic minority	11.45 (2.66)	−0.99	−6.51, 4.51	0.22	−5.84, 6.28
Employment status					
Working or retired	13.44 (0.59)	1.00		1.00	
Not working	10.77 (0.72)	−2.67	−5.45, 0.11	−2.02	−4.95, 0.90
Housing status					
Fixed address	12.66 (0.49)	1.00		1.00	
No fixed address	9.23 (1.55)	−3.43	−6.90, 0.04	−2.78	−6.41, 0.85
Rank					
Officer	13.74 (1.36)	1.00		1.00	
Other rank	12.24 (0.49)	−1.50	−4.88, 1.89	−1.73	−4.86, 1.39
Early service leaver					
No (>4 years’ service)	12.53 (0.48)	1.00		1.00	
Yes (<4 years’ service)	12.19 (2.04)	−0.34	−3.22, 2.54	0.97	−2.08, 4.03
Reason for leaving					
Voluntary	13.29 (0.63)	1.00		1.00	
Non-Voluntary	11.34 (0.70)	−1.95	−4.51, 0.61	-1.22	−4.30, 1.85

*Notes:* Reason for leaving non-voluntary also includes medical discharge.

^*^ *P* < 0.05,

^**^ *P* < 0.01.

A second multiple linear regression model was run to investigate associations between probable insomnia disorder and functional outcomes. Table 3 presents the outcome in more detail. No factors were significantly associated within the adjusted model. It was found in the unadjusted model that those who reported low social support (−2.72, 95% CI −4.38, −1.06, *P* < 0.01) and loneliness (−2.43, 95% CI −3.79, −1.07, *P* < 0.01) were significantly associated with probable insomnia disorder. Again, these did not remain significant in the adjusted model.

**Table 3:** Associations between functional outcomes and sleep

	Mean (SE)	Unadjusted	95% Confidence Interval	Adjusted	95% Confidence Interval
Social support					
No	14.40 (0.40)	1.00		1.00	
Yes	11.68 (0.57)	−2.72 ^**^	−4.38, −1.06	−1.35	−2.41, −0.30
Loneliness					
No	14.42 (0.92)	1.00		1.00	
Yes	11.99 (0.55)	−2.43 ^**^	−3.79, −1.07	−2.61	−4.62, −0.59
Well-being					
No	13.56 (0.75)	1.00		1.00	
Yes	11.85 (0.59)	−1.71	−4.34, 0.92	−0.66	−2.80, 1.48
Alcohol use					
No	12.96 (0.61)	1.00		1.00	
Yes	11.26 (1.07)	−1.70	−3.66, 0.26	−1.24	−2.70, 0.21

Notes: Social support: OSSS-3 = Oslo Social Support Scale; Loneliness: UCLA-3 = UCLA loneliness scale; Well-being: Short Warwick–Edinburgh Mental Well-Being Scale (SWENWBS); Alcohol misuse: AUDIT = Alcohol Use Disorder Identification Test.

^**^ *P < *0.01.

A final multiple linear regression model was run to look at the relationship between health outcomes and sleep. Table 4 presents the analysis in more detail. Those with physical health problems had a significant association with sleep problems (−3.39, 95% CI −4.67, −2.11, *P* < 0.001). It was also found that difficulties with anger were significantly associated with sleep (1.26, 95% CI 0.34, 2.18, *P* < 0.05). Within the unadjusted model, it was also found that CPTSD (−2.18, 95% CI −3.86, −0.50, *P* <* *0.05), obsessive–compulsive disorder (OCD; −3.58, 95% CI −6.16, −1.01, *P < *0.05) and CMD (−3.28, 95% CI −4.57, −2.00, *P* <* *0.05) were significantly associated with sleep difficulties; however, this did not remain within the adjusted model.

**Table 4:** Associations between health outcomes and sleep

	Mean (SE)	Unadjusted	95% Confidence Interval	Adjusted	95% Confidence Interval
CMD					
No	15.08 (0.97)	1.00		1.00	
Yes	11.80 (0.53)	−3.28 ^*^	−4.57, −2.00	−1.79	−3.81,0.24
Anger difficulties					
No	12.69 (0.58)	1.00		1.00	
Yes	12.32 (0.84)	−0.37	−2.01, 1.28	1.26^*^	0.34, 2.18
CPTSD					
No	13.85 (0.65)	1.00		1.00	
Yes	11.67 (0.66)	−2.18^*^	−3.86, −0.50	-1.13	−3.51, 1.26
OCD					
No	12.33 (0.54)	1.00		1.00	
Yes	8.75 (1.12)	−3.58 ^*^	−6.16, −1.01	−1.85	−3.88, 0.18
Physical health					
No	13.74 (0.55)	1.00		1.00	
Yes	9.93 (0.80)	−3.81^***^	−5.43, −2.18	-3.39^***^	−4.67, −2.11

*Notes*. CMD (Common Mental Health Difficulties): GHQ-12 = General Health Questionnaire; Anger Difficulties: DAR-5 = Dimensions of Anger Reactions; CPTSD: ITQ = International Trauma Questionnaire; OCD: Y-BOCS = Yale–Brown Obsessive Compulsive Scale; Physical health difficulties: PHQ-15 = Patient Health Questionnaire.

^***^ *P <* 0.001;

^*^ *P < *0.05.

## Discussion

Within our clinical sample, the majority (70%) reported a possible insomnia disorder. This highlights the clinical importance to investigate the prevalence of sleep disorders in this population. Multiple liner regression models found that those with physical health problems were significantly more likely to have probable insomnia disorder, as well as those aged between 35 and 44.

Insomnia disorder can have consequences in both the night (trouble falling or staying asleep) and daytime (fatigue and sleepiness) [3] [as well as implications like increased risk of accidents, absenteeism and a lower quality of life [6]. The high prevalence within treatment-seeking veterans suggests they could be a vulnerable population to insomnia disorders, and a vast majority of our sample is at risk of implications such as these.

The adjusted regression model found that physical health problems were significantly associated with a probable insomnia disorder. This has been found in previous research [9, 12]; however, as this study is cross-sectional, we are unable to determine causality between these two factors. This suggests those who are presenting with poor physical health may also have an underlying insomnia disorder, and targeted treatments for both disorders may be beneficial.

Additionally, difficulties with anger was significantly associated with sleep in the adjusted model but no association was found in the unadjusted model. Due to modest differences in mean scores on the SCI, this may be explained by anger being assoicated with another outcome. It was found that several health outcomes including CMDs, OCD and CPTSD were significantly associated with probable insomnia disorder in the unadjusted linear regression model; however, this was not maintained in the adjusted model. This is consistent with both previous literature and with the symptom profile of disorders such as CMDs and CPTSD whereby sleep disorders are a frequent symptom. [10, 12, 14, 29, 30].

One method to reduce the prevalence of insomnia disorder in veterans could be earlier intervention, such as by promoting the importance of maintaining a healthy sleep pattern among serving Armed Forces personnel, while balancing it with the operational demands of deployment and teaching how to maintain this once leaving the Armed Forces. Current UK guidance states probable insomnia disorder should be managed by their general practitioner or family doctor (GP) [2]. Our findings suggest the importance of other clinical services treating veterans also assessing for probable insomnia disorder and then providing support to also improve sleep.

Some interventions have been successful in treating insomnia disorder such as Cognitive Behaviour Therapy for Insomnia (CBT-I). The USA currently uses CBT-I as a first-line intervention for the treatment of insomnia disorder in the military. However, a recent meta-analysis revealed inconsistent findings with the use of CBT-I within veteran samples and therefore may need further clinical trials before it is a recommended treatment within the UK for veterans [31].

Furthermore, data from the USA have shown that some Armed Forces personnel feel there are barriers to reaching out for help due to a stigma within military culture surrounding having a greater need for sleep [10]. This stigma could be due to ideas that sleep problems could have severe negative career consequences or require intensive treatment to solve [9]. Research in the UK would need to address whether the same barriers to care are experienced regarding probable insomnia disorder.

However, this study has several limitations. First, some populations may have been over- and under-represented and may lack generalizability. Most of the sample were male (96%), had served in the British Army (82%) and were White (95%). Moreover, the entire sample was treatment-seeking veterans. Due to previous research finding that insomnia disorder is often co-morbid with mental and physical health conditions [14], the prevalence rate found in this study may be over-estimated, and may not be representative of the wider, non-treatment-seeking veteran community.

Data from this study did not include factors such as combat exposure or deployment history. There has been mixed literature surrounding the association between deployment and combat with probable insomnia disorder [9] but future research could aim to use these variables to examine if it influences the results. We adopted an analysis technique as *a priori* to explore analysis separately (multi linear regression); however, another method like a stepwise technique may have been better.

This study is also limited as despite finding that those aged 35–44 were significantly more likely to report probable insomnia disorder, previous research has found older adults tend to be associated with insomnia disorder [6]. By having age as a continuous variable, we may have been able to explore associations with age in more depth, a technique future studies could adopt.

The data from this study were collected between August and October 2020, during the COVID-19 pandemic. Overall, this study received a good response rate of 43% and is comparable to other veteran studies whereby data were collected in the COVID-19 period such as Sharp et al. [32] (44%). While there was no National Lockdown during this period, it is possible that the effects of the pandemic could have affected the findings. Hence, more research outside of the COVID-19 pandemic is needed to confirm the potential effects and prevalence of insomnia disorder in veterans.

Future research could look at more in-depth types of sleep disorders within this population as this paper only identified a probable insomnia disorder. While this is a highly prevalent type of sleep disorder [31], some research in USA serving military has shown frequencies with other disorders such as sleep apnoea and snoring [29]. However, these studies tend to rely on more invasive methods such as wearing measuring devices during sleep meaning there are caveats compared to self-report data regarding the ease of data collection.

In conclusion, this study found that probable insomnia disorder is highly prevalent amongst UK treatment-seeking veterans. Moreover, associations between probable insomnia disorder and socio-demographic, military, functional and health factors were discussed. There are several limitations to consider when interpreting these findings, however, such as being restricted to treatment-seeking veterans. Nonetheless, this paper has contributed to a very limited amount of literature regarding probable insomnia in UK veterans and presents potential paths for future research. This encourages the need for developing the knowledge of interventions such as CBT-I within the UK veteran population, which could have significant clinical implications.

## References

[1] Banks S , Dinges DF. Behavioral and physiological consequences of sleep restriction. J Clin Sleep Med 2007;3:519–528. 17803017 PMC1978335

[2] Ministry of Defence. UK Armed Forces Mental Health: Annual Summary & Trends Over Time, 2007/08–2019/20. London: Ministry of Defence; 2020.

[3] WHO. ICD-11 for Mortality and Morbidity Statistics (Version: 01/2023). Geneva, Switzerland: World Health Organization, 2019/2023.

[4] Alexander M , Ray MA, Hébert JR et al. The national veteran sleep disorder study: descriptive epidemiology and secular trends, 2000–2010. Sleep 2016;39:1399–1410. 27091538 10.5665/sleep.5972 PMC4909622

[5] Calem M , Bisla J, Begum A et al. Increased prevalence of insomnia and changes in hypnotics use in England over 15 years: analysis of the 1993, 2000, and 2007 National Psychiatric Morbidity Surveys. Sleep 2012;35:377–384. 22379244 10.5665/sleep.1700 PMC3274339

[6] Morphy H , Dunn KM, Lewis M, Boardman HF, Croft PR. Epidemiology of insomnia: a longitudinal study in a UK population. Sleep 2007;30:274–280. 17425223

[7] Wilson S , Anderson K, Baldwin D et al. British Association for Psychopharmacology consensus statement on evidence-based treatment of insomnia, parasomnias and circadian rhythm disorders: an update. J Psychopharmacol 2019;33:923–947. 31271339 10.1177/0269881119855343

[8] Plumb TR , Peachey JT, Zelman DC. Sleep disturbance is common among service members and veterans of Operations Enduring Freedom and Iraqi Freedom. Psychol Serv 2014;11:209–219. 24274111 10.1037/a0034958

[9] Troxel WM , Shih RA, Pedersen E et al. Barriers to achieving healthy sleep among service members. In: *Sleep in the Military.* Promoting Healthy Sleep Among U.S. Service Members. RAND Corporation, 2015:101–116.

[10] Troxel WM , Shih RA, Pedersen ER et al. Sleep in the military: Promoting healthy sleep among US service members. *Rand Health Q* 2015;5:19. 10.7249/rhq-05-02-19 PMC5158299 28083395

[11] Riemann D , Berger M, Voderholzer U. Sleep and depression—results from psychobiological studies: an overview. Biol Psychol 2001;57:67–103. 11454435 10.1016/s0301-0511(01)00090-4

[12] Bramoweth AD , Germain A. Deployment-related insomnia in military personnel and veterans. Curr Psychiatry Rep 2013;15:1–8. 10.1007/s11920-013-0401-4 PMC3832138 24005883

[13] Stewart R , Besset A, Bebbington P et al. Insomnia comorbidity and impact and hypnotic use by age group in a national survey population aged 16 to 74 years. Sleep 2006;29:1391–1397. 17162985 10.1093/sleep/29.11.1391

[14] Hunt E , Greenberg N, Jones N. Poor sleep after military deployment: associations with mental health difficulties. Occup Med (Oxf) 2016;66:669–675. 27543646 10.1093/occmed/kqw116

[15] Gangwisch JE , Heymsfield SB, Boden-Albala B et al. Sleep duration as a risk factor for diabetes incidence in a large US sample. Sleep 2007;30:1667–1673. 18246976 10.1093/sleep/30.12.1667 PMC2276127

[16] Espie CA , Kyle SD, Hames P, Gardani M, Fleming L, Cape J. The Sleep Condition Indicator: a clinical screening tool to evaluate insomnia disorder. BMJ Open 2014;4:e004183. 10.1136/bmjopen-2013-004183 PMC3964344 24643168

[17] Hughes ME , Waite LJ, Hawkley LC, Cacioppo JT. A short scale for measuring loneliness in large surveys: results from two population-based studies. Res Aging 2004;26:655–672. 18504506 10.1177/0164027504268574 PMC2394670

[18] Dalgard O. Community health profile as tool for psychiatric prevention. Promot Mental Health 1996;5:681–695.

[19] Tennant R , Hiller L, Fishwick R et al. The Warwick-Edinburgh mental well-being scale (WEMWBS): development and UK validation. Health Qual Life Outcomes 2007;5:1–13. 18042300 10.1186/1477-7525-5-63 PMC2222612

[20] Spitzer RL , Kroenke K, Williams JB, Group PHQPCS, Group PHQPCS. Validation and utility of a self-report version of PRIME-MD: the PHQ primary care study. JAMA 1999;282:1737–1744. 10568646 10.1001/jama.282.18.1737

[21] Goldberg DP. User’s Guide to the General Health Questionnaire. Windsor: NFER-Nelson,1988.

[22] Saunders JB , Aasland OG, Babor TF, De La Fuente JR, Grant M. Development of the alcohol use disorders identification test (AUDIT): WHO collaborative project on early detection of persons with harmful alcohol consumption‐II. Addiction 1993;88:791–804. 8329970 10.1111/j.1360-0443.1993.tb02093.x

[23] Goodman WK , Price LH, Rasmussen SA et al. The Yale-Brown obsessive compulsive scale: I. Development, use, and reliability. Arch Gen Psychiatry 1989;46:1006–1011. 2684084 10.1001/archpsyc.1989.01810110048007

[24] Forbes D , Alkemade N, Hopcraft D et al. Evaluation of the Dimensions of Anger Reactions-5 (DAR-5) Scale in combat veterans with posttraumatic stress disorder. J Anxiety Disord 2014;28:830–835. 25445072 10.1016/j.janxdis.2014.09.015

[25] Cloitre M , Shevlin M, Brewin CR et al. The International Trauma Questionnaire: development of a self‐report measure of ICD‐11 PTSD and complex PTSD. Acta Psychiatr Scand 2018;138:536–546. 30178492 10.1111/acps.12956

[26] Murphy D , Shevlin M, Pearson E et al. A validation study of the International Trauma Questionnaire to assess post-traumatic stress disorder in treatment-seeking veterans. Br J Psychiatry 2020;216:132–137. 32345413 10.1192/bjp.2020.9

[27] Williamson C , Baumann J, Murphy D. Exploring the health and well-being of a national sample of UK treatment-seeking veterans. Psychol Trauma Theory Res Pract Policy 2023;15:672–680. 10.1037/tra0001356 36222665

[28] StataCorp. Stata Statistical Software: Release 13. College Station, TX: StataCorp LP.; 2013.

[29] Mysliwiec V , McGraw L, Pierce R, Smith P, Trapp B, Roth BJ. Sleep disorders and associated medical comorbidities in active duty military personnel. Sleep 2013;36:167–174. 23372263 10.5665/sleep.2364 PMC3543057

[30] Caldwell JA , Knapik JJ, Shing TL, Kardouni JR, Lieberman HR. The association of insomnia and sleep apnea with deployment and combat exposure in the entire population of US army soldiers from 1997 to 2011: a retrospective cohort investigation. Sleep 2019;42:zsz112. 31106808 10.1093/sleep/zsz112 PMC6685319

[31] Rigley J , Neilson C, Murphy D, Watson F. How effectively does CBT-I address the traumatic and functional causes of insomnia and sleep disturbance in veterans? J Mil Veteran Fam Health 2022;8:6–18.

[32] Sharp M-L , Serfioti D, Jones M et al. UK veterans’ mental health and well-being before and during the COVID-19 pandemic: a longitudinal cohort study. BMJ Open 2021;11:e049815. 10.1136/bmjopen-2021-049815 PMC8406464 34452965

